# Controlling Stimulated Emission via Intramolecular Charge Transfer in Amino-Coumarin Dyes: Switching from Reverse Saturable to Saturable Absorption

**DOI:** 10.3390/molecules30183799

**Published:** 2025-09-18

**Authors:** Jidong Jia, Siya Wu, Yinlin Lu, Jingyuan Xu, Hang Zuo, Xingzhi Wu, Yinglin Song

**Affiliations:** 1 School of Electronic Information, Huzhou College, Huzhou 313000, China; 18200450439@163.com; 2 Department of Physics, Soochow University, Suzhou 215123, China; 20234208046@stu.suda.edu.cn (Y.L.); 20234208069@stu.suda.edu.cn (J.X.); 3 Huzhou Science and Technology Information Research Institute, Huzhou 313000, China; zucker_han@126.com; 4 Jiangsu Key Laboratory of Micro and Nano Heat Fluid Flow Technology and Energy Application, School of Physical Science and Technology, Suzhou University of Science and Technology, Suzhou 215009, China

**Keywords:** nonlinear optical absorption, excited state dynamics, aminocoumarin dyes, intramolecular charge transfer

## Abstract

Given the pivotal role of coumarins as tunable nonlinear optical (NLO) materials for advanced photonics, this study aims to decipher the regulatory mechanisms governing their excited-state dynamics and nonlinear absorption. In this study, two amino-coumarin dyes (102 and 153) differing in electron-withdrawing groups are synthesized to probe the impact of intramolecular charge transfer (ICT) on transient dynamics and nonlinear absorption. Frontier orbital and natural transition orbital analyses reveal subtle alterations in the ICT characteristics of amino-coumarin molecules. These minor modifications induce a significant red shift in the stimulated emission band within transient absorption spectroscopy, ultimately triggering a transition from reverse saturable absorption (RSA) to saturable absorption (SA) at 515 nm. Our findings demonstrate that, with straightforward molecular modifications, coumarins emerge as promising dual-function materials for saturable absorption and optical limiting.

## 1. Introduction

Nonlinear optical absorption (NLA) comprises saturable absorption (SA) and reverse saturable absorption (RSA), governing saturable absorbers (SAs) and optical limiters (OLs). SAs leverage intensity-dependent transparency (α∝1/*I*) for femtosecond pulse compression in mode-locked lasers, passive Q-switching, and optical modulation [1,2,3]. OLs utilize RSA-induced attenuation (α∝*I*) for laser protection, shielding eyes/sensors against high-energy beams with >90% suppression [4,5,6]. Coumarin molecules demonstrate the potential for application in both SAs and OLs, making them a focus of current research [7,8,9,10].

Coumarin derivatives are cornerstone materials in nonlinear optics (NLO), valued for their readily tunable π-conjugated scaffolds and exceptional optoelectronic properties. These attributes support their critical applications in advanced photonic technologies, such as optical limiting for laser protection, efficient frequency conversion devices, deep-tissue multiphoton bioimaging, and targeted photodynamic therapy [11,12,13,14,15,16]. While structure–property relationships governing static NLO responses (e.g., hyperpolarizabilities) have been extensively explored, the fundamental understanding of dynamic processes—specifically, how ultrafast excited-state dynamics dictate and can be manipulated to control nonlinear optical absorption—remains comparatively less elucidated [17,18,19,20,21,22]. Consequently, this study aims to elucidate the regulatory mechanisms governing excited-state dynamics and NLA in tailored coumarin systems.

**Figure 1:**
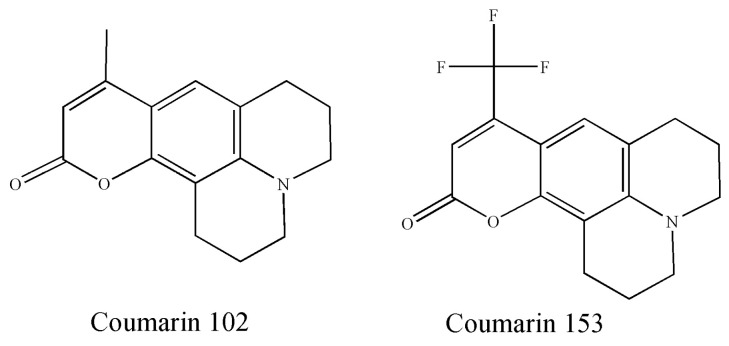
Molecular structures of coumarin 102 and 153.

To investigate the effect of ICT on the excited-state dynamics and the NLA properties of coumarins while minimizing interference from other factors, a molecular design strategy employing as simple a structure as possible is essential. Accordingly, we designed and synthesized two amino-coumarin derivatives, 102 and 153, featuring highly similar molecular frameworks (Figure 1). Both molecules possess the same julolidine donor substituent at the C7 position of the 1,2-benzopyrone core but differ at the C4 position, bearing methyl (CH_3_) and trifluoromethyl (CF_3_) groups, respectively. The incorporation of a trifluoromethyl (CF_3_) group in coumarin 153 enhances the electron-withdrawing ability of the 1,2-benzopyrone moiety, thereby improving the intramolecular charge transfer (ICT) performance.

This study employs frontier molecular orbital (FMO) and natural transition orbital (NTO) analyses to characterize the ICT performance [23,24,25,26,27,28,29,30]. To validate the reliability of our excited-state calculations, we simulated the UV-vis absorption spectra of coumarin derivatives and observed excellent agreement with the experimental data. Moreover, we analyzed the modulation of ICT on stimulated emission bands and NLA using transient absorption spectroscopy (TAS) and the Z-scan technique, respectively. Our research demonstrates that even a modest enhancement of ICT in coumarin systems induces a significant red shift in the stimulated emission band, driving the transition from RSA to SA in nonlinear absorption. This demonstrates coumarins’ dual functionality as saturable absorbers and optical limiters based on their switchable nonlinear absorption.

## 2. Results and Discussion

### 2.1. Molecular Design and Electronic Transition Analysis

The strategic selection of methyl (CH_3_) and trifluoromethyl (CF_3_) groups was guided by a key molecular design principle: to isolate the effect of electronic perturbation while minimizing steric contributions. Despite their opposing electronic character—CH_3_ being weakly electron-donating and CF_3_ strongly electron-withdrawing—these groups possess a comparable steric bulk. This ensures that differences in photophysical properties arise primarily from electronic effects rather than structural changes.

**Figure 2:**
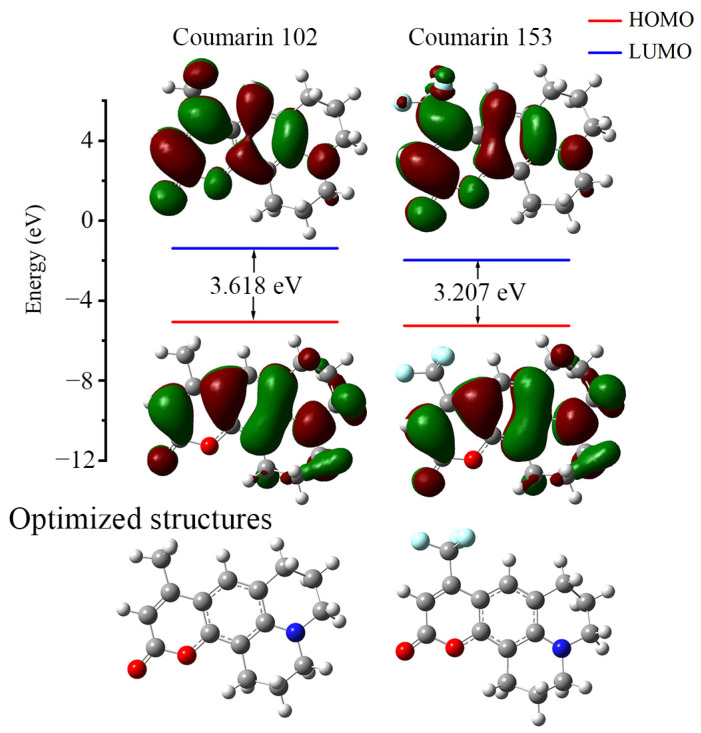
FMOs of coumarin 102 and 153.

Energy offsets between HOMO and LUMO orbitals in coumarin 102/153 (Figure 2) quantify their electron transition efficiencies. The highly congruent molecular geometries result in virtually indistinguishable HOMO orbital distributions. The LUMO, however, shows markedly different spatial distributions: it predominantly localizes on either the C4-methyl or C4-trifluoromethyl peripheral substituents. Owing to CF_3_’s superior electron-withdrawing strength over CH_3_, coumarin 153 shows enhanced ICT and a reduced HOMO-LUMO gap (3.207 eV vs. 3.618 eV for coumarin 102). Overall, the HOMO → LUMO transitions in both coumarin 102 and 153 are primarily local excitations with minor ICT contributions.

**Figure 3:**
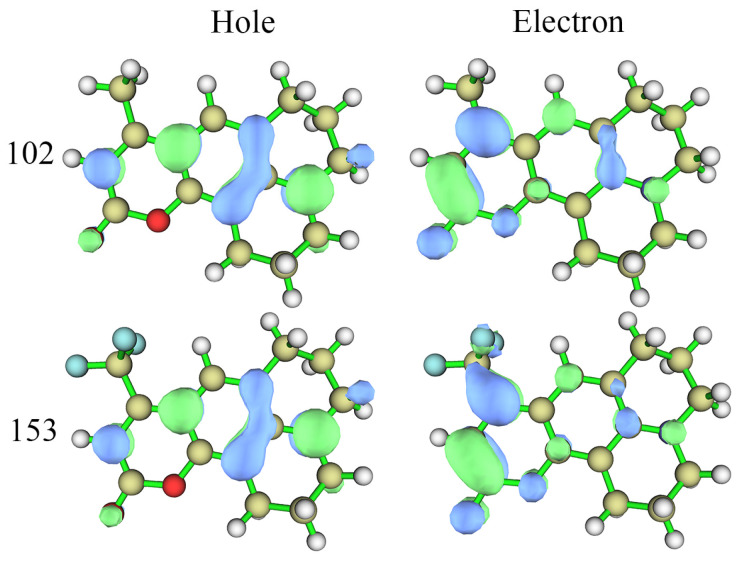
NTOs of coumarin 102 and 153 in the excited state S_1_.

The NTO analysis reveals an enhanced ICT in the S_1_ state of coumarin 153 more intuitively—see Figure 3. In both compounds, the hole is delocalized over the molecular framework, whereas the electron predominantly localizes on the 1,2-benzopyrone acceptor moiety. Thus, the S_0_ → S_1_ electronic transition exhibits dual characteristics: local excitation and ICT. Critically, introducing the CF_3_ group into coumarin 153 significantly enhances its ICT character. This effect correlates with distinct electron density localization on the C4-trifluoromethyl substituent—a feature absent at the C4-methyl site in coumarin 102.

To characterize the ICT, we evaluated the dipole moments of the ground (S_0_) and excited (S_1_) states, as well as the transition dipole moment (Table 1). The difference in dipole moments between the ground and excited states (Δμ = |μ_11_ − μ_00_|) is as follows: a larger Δμ indicates a greater redistribution of electron density upon excitation, reflecting stronger ICT. For Coumarins 102 and 153, the calculated Δμ values are 4.27 and 5.23 a.u., respectively, suggesting a stronger ICT in coumarin 153 than in coumarin 102. Additionally, the static polarizability and second-order static hyperpolarizability of coumarin 102 and 153 were computed using the sum-over-states method, incorporating 50 excited states (Table 1). The results indicate that the enhanced ICT in coumarin 153 leads to a significant increase in both its static polarizability and second-order static hyperpolarizability.

Our computational results validate this approach and directly support our hypothesis. The excited-state dipole moment (µ_11_) is significantly larger in coumarin 153 (CF_3_), indicating a more polarized excited state and strengthened ICT character. Correspondingly, the second-order hyperpolarizability (γ) of coumarin 153 is 1.6 times greater than that of coumarin 102, demonstrating the CF_3_ group’s enhanced ability to modulate electron conjugation and nonlinear optical response.

### 2.2. UV-Vis Absorption and Fluorescence Emission

**Figure 4:**
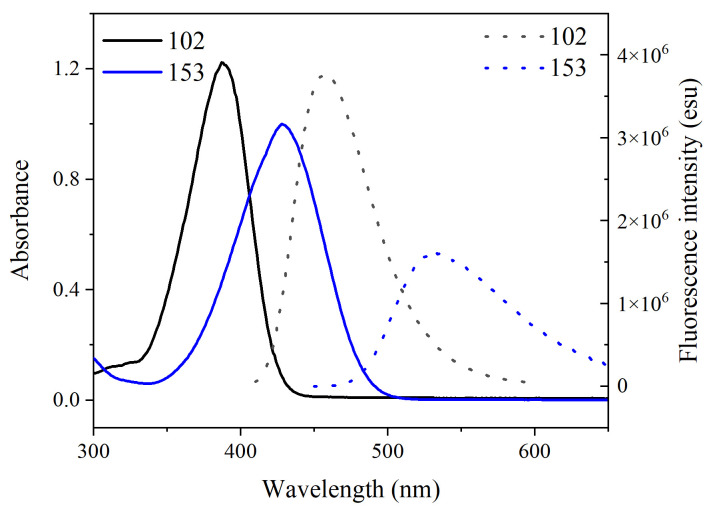
UV-vis absorption spectra and fluorescence emission spectra of coumarin 102 and 153 in dimethyl sulfoxide (DMSO).

The UV-Vis absorption and fluorescence emission spectra of coumarin 102 and 153 were recorded to investigate their excited-state energy levels—see Figure 4. Coumarin 102 and 153 exhibit maximum absorption peaks at 387 nm and 426 nm and maximum emission peaks at 458 nm and 532 nm, respectively. The significant red shift observed in both the absorption and emission bands of coumarin 153, compared to coumarin 102, aligns with electronic transition analysis. The smaller HOMO–LUMO gap in coumarin 153 (3.207 eV vs. 3.618 eV in coumarin 102) originates from the stronger electron-withdrawing effect of the CF_3_ group, which significantly stabilizes the LUMO energy level and enhances the charge transfer character. According to the Planck–Einstein relation (E=hc / λ), a reduced energy gap ΔE corresponds directly to a longer wavelength λ of the absorbed or emitted photon. Thus, the narrower gap in coumarin 153 rationalizes the observed redshift in both absorption (426 nm vs. 387 nm) and emission (532 nm vs. 458 nm) relative to coumarin 102. This provides a direct link between electronic structure modulation and spectral properties through enhanced ICT. We performed fluorescence decay experiments to investigate the dynamic evolution, as shown in Figure S1 (Supporting Information).

**Figure 5:**
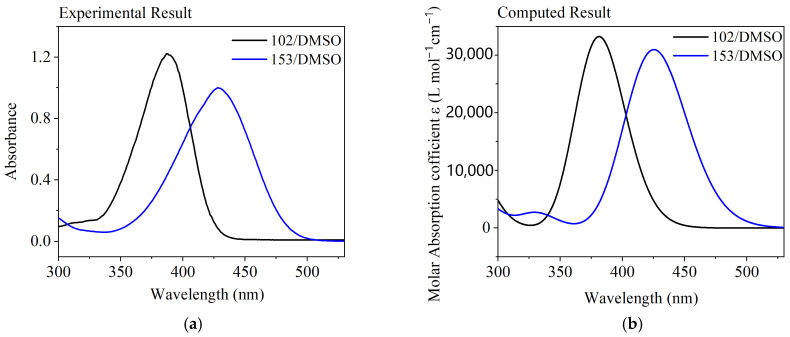
Comparison of measured and simulated UV-Vis absorption spectra: (**a**) measured; (**b**) simulated.

To further verify the reliability of the excited-state calculations, the simulated UV-Vis absorption spectra were compared with experimental data; good agreement is shown in Figure 5. The simulated and experimental spectra exhibit closely matching shapes. With deviations of only 6 nm and 1 nm from experimental values (387 nm vs. 381 nm for coumarin 102; 426 nm vs. 425 nm for coumarin 153), the simulated absorption peaks confirm the reliability of our computational approach. The NTO analysis (Figure 3) confirms that the key absorption features (387 nm for C102 and 426 nm for C153) are primarily due to a singlet π → π* transition, an assignment further supported by the agreement with the simulated spectra (Figure 5b).

### 2.3. Excited-State Dynamics Analysis

**Figure 6:**
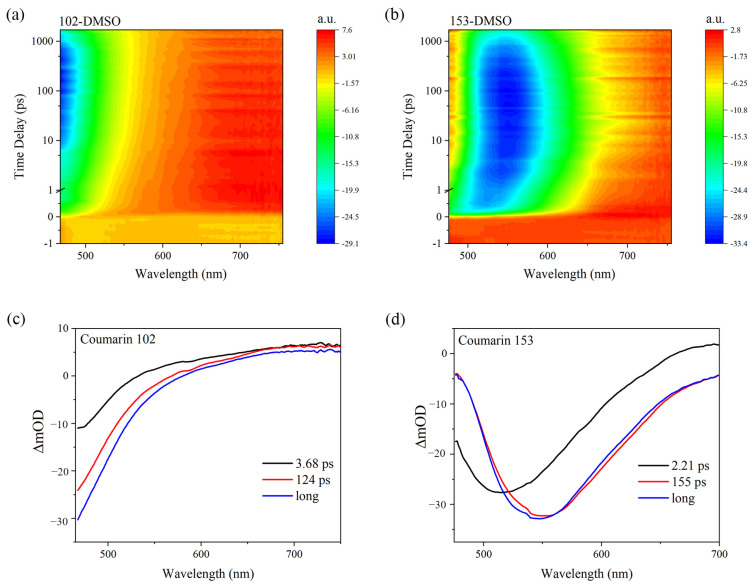
Excited state dynamics analysis of coumarin 102 and 153: (**a**,**b**) TAS of coumarin 102/153 in DMSO; (**c**,**d**) EADS of coumarin 102/153.

To investigate the effect of ICT on the transient dynamics of coumarins—including the stimulated emission and excited-state absorption bands—we recorded the TAS of coumarins 102 and 153 in DMSO, as shown in Figure 6a,b. The transient absorption spectra (TAS) of coumarins 102 and 153 reveal two distinct features: (1) stimulated emission bands (negative signals; blue/green/yellow regions); (2) excited-state absorption bands (positive signals; red regions). Coumarin 153 shows significantly red-shifted stimulated and fluorescence emission bands versus coumarin 102, attributable to the enhanced ICT. Global fitting analysis was performed [31], with the resulting evolution-associated difference spectra (EADS) shown in Figure 6c,d. The enhanced ICT strength of coumarin 153 results in a significant red shift in its spectral features at 155 ps. In contrast, the weaker ICT character of coumarin 102 explains the absence of a pronounced red shift in its dynamic evolution (124 ps).

**Figure 7:**
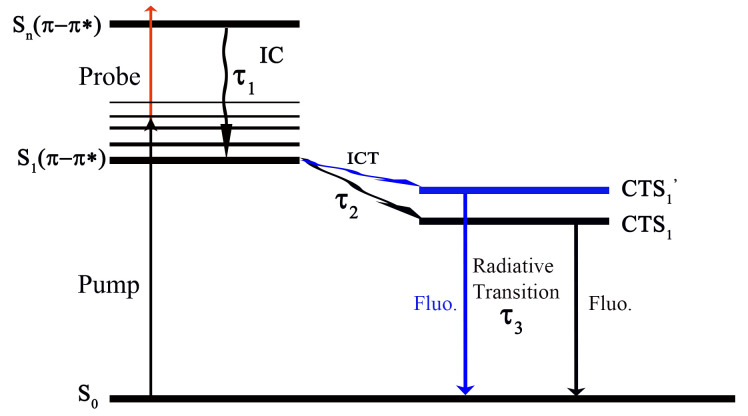
Electron relaxation model.

An electron relaxation model was developed to describe the relaxation dynamics (Figure 7). Upon photoexcitation to the high-energy S_n_(π − π*) state by a pump pulse and a probe pulse, the system undergoes ultrafast internal conversion (IC) to the lowest excited singlet state S_1_(π − π*). From S_1_, ICT generates a charge-transfer singlet state (CTS_1_). This CTS_1_ state decays radiatively to the ground state, producing fluorescence. Transient species along this pathway (e.g., IC, CTS_1_) can be monitored through probe pulse absorption, revealing the multi-stage relaxation dynamics.

### 2.4. Tunable Nonlinear Optical Absorption

**Figure 8:**
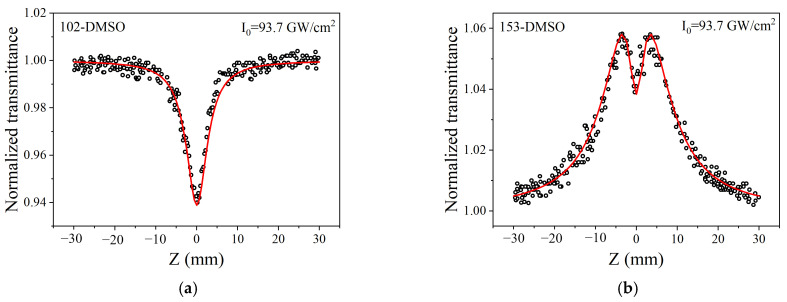
Femtosecond Z-scan experiments at 515 nm: (**a**) RSA of coumarin 102; (**b**) SA of coumarin 153.

Femtosecond Z-scan measurements at 515 nm (Figure 8) confirm that nonlinear absorption conversion is tunable via stimulated emission band modulation. This excitation wavelength was selected based on the TAS results (Figure 6a,b). The TAS data reveal that 515 nm coincides with the band-edge of the stimulated emission band for coumarin 102. In contrast, coumarin 153 exhibits a strongly red-shifted stimulated emission band due to its enhanced ICT character. As a result, the same 515 nm excitation wavelength falls near the center of the stimulated emission band in coumarin 153, enabling us to investigate the distinctly different nonlinear optical responses arising from their differing ICT strengths. In Z-scan experiments, the sample is translated along the optical axis through the focal plane via a motorized linear stage. The light intensity peaks at focus and declines radially. Materials showing the intensity-dependent absorption enhancement follow RSA behavior, whereas those with absorption reduction exhibit SA. In RSA behavior, transmittance decreases progressively near focus, manifesting as a valley in Z-scan traces. SA exhibits its characteristic signature through increasing transmittance near focus, manifesting as a pronounced peak in Z-scan measurements.

The observed RSA in coumarin 102 (Figure 8a) follows $\alpha =\alpha_{0}+\beta I$ (*β* > 0), where *α*_0_ is the linear absorption coefficient, *β* > 0 is the nonlinear absorption coefficient, and *I* denotes the laser intensity. It is observed that, under an input intensity of 93.7 GW/cm^2^, coumarin 153 exhibits clear SA behavior. Notably, however, a significant decrease in normalized transmittance occurs at the focal point. This suggests the presence of a competitive process between SA and RSA. The optical response transitions from SA to RSA as the intensity increases. This occurs because the stimulated emission, which governs at low intensities, is superseded by dominant excited-state absorption at high intensities. Therefore, the total absorption coefficient α of coumarin 153 can be expressed as

$$\alpha =\frac{\alpha_{0}}{1+\frac{I}{I_{c}}}+\beta I,$$

where *I_c_* denotes the saturation light intensity. We numerically fitted the experimental data, with parameters summarized in Table 2.

Overall, femtosecond Z-scans at 515 nm capture ICT-driven RSA/SA conversion in coumarins, governed by modulated stimulated emission dynamics. As previously explained, for coumarin 102, 515 nm is within its ESA band but on the tail of its weak SE band. The dominant ESA over the stimulated emission leads to net RSA. For coumarin 153, the red-shifted spectrum means 515 nm now coincides with the peak of its intense stimulated emission (SE) band. This creates a condition where the stimulated emission dominates over ESA at this wavelength. Upon excitation, a large population in the first excited state (S_1_) leads to a dominant SE process, which amplifies the transmitted light and results in the overall SA behavior observed in the open-aperture Z-scan curve. The faint dip observed at the focus of the Z-scan trace for coumarin 153 is a critical signature of the residual competition from ESA. At the very highest laser fluences at the focus, the ESA process becomes non-negligible and momentarily competes with the dominant SE process, causing a slight decrease in net transmission before the SA effect prevails again as the beam defocuses. This nuanced spectral overlap and competition between SE and ESA at 515 nm is a direct consequence of the red-shifted energy levels induced by the strong ICT character of the trifluoromethyl group.

## 3. Materials and Methods

### 3.1. Materials

Coumarin 102 (Macklin, CAS 41267-76-9) and coumarin 153 (Macklin, CAS 53518-18-6) were purchased from Macklin Reagent Company (Shanghai, China) and used without further purification. Detailed synthetic protocols are elaborated in the Supporting Information (Section 3).

### 3.2. Quantum Chemical Calculation

Using Gaussian 09 software, all calculations were performed at the B3LYP/6-311++G(2d,2p) level: geometry optimizations with density functional theory (DFT) and excited-state calculations with time-dependent density functional theory (TD-DFT) [32,33,34,35]. Solvent effects (DMSO) were incorporated using the SMD implicit solvation model [36]. Multiwfn 3.8 software was employed for NTO analysis of S_0_ → S_1_ electron transitions and simulation of UV-vis absorption spectra [37,38].

### 3.3. Nonlinear Optical Experiments

Both TAS and Z-scan experiments used a femtosecond laser system comprising a Yb:KGW laser-amplifier (Light Conversion PHAROS-SP, Vilnius, Lithuania; 1030 nm, 190 fs, 6 kHz) and an optical parametric amplifier (OPA, Light Conversion ORPHEUS) [39,40]. TAS utilized 380 nm pump pulses (OPA-generated, 6 kHz) and a VIS-NIR probe from sapphire-based supercontinuum generation driven by femtosecond pulses. The laser source for the transient absorption spectroscopy (TAS) experiments operated at a fundamental repetition rate of 6 kHz. However, to prevent any cumulative thermal effects on the measurements, a mechanical chopper was used to synchronously reduce the effective repetition rate of the pump beam to 1 kHz. This effective rate is the most relevant parameter for the collected data and ensures that the sample fully recovers between excitation pulses. Z-scan measurements used OPA-generated 515 nm excitation at a 20 Hz repetition rate to minimize thermally induced nonlinearities.

All samples (coumarin 102 and 153) were prepared at a concentration of 5 mM in anhydrous dimethyl sulfoxide (DMSO, Sigma-Aldrich, St. Louis, MO, USA, ≥99.9%). DMSO was selected as the solvent for two primary reasons: (1) It provides high solubility for both coumarin derivatives, ensuring the formation of stable, homogeneous solutions required for both Z-scan and TAS measurements; and (2) its low volatility prevents solvent evaporation and consequent concentration changes during the extended duration of the experiments, thereby guaranteeing solution stability. The mixtures were subjected to ultrasonic treatment in an ultrasonic bath for approximately 10–15 min until complete dissolution was achieved, yielding clear, homogeneous solutions. To prevent any potential photo-degradation, the prepared solutions were stored in amber vials and kept in the dark at room temperature when not in use. The integrity of the samples was confirmed by comparing the UV-Vis absorption spectra before and after the measurement series, and no significant changes were observed.

Coumarin 102 and 153 solutions in DMSO (5 mM) were examined in 2 mm path length quartz cuvettes for both TAS and Z-scan experiments. Moreover, the solvent exhibited no significant nonlinear optical response, so all observed responses were attributed to the solute. DMSO was selected primarily because coumarin 102 and 153 exhibit excellent solubility in this solvent, which is essential to achieve the high sample concentrations required for our Z-scan and TAS measurements. Furthermore, DMSO is non-volatile and maintains a stable concentration throughout the measurement period, making it a robust and reliable choice for these sensitive experiments.

## 4. Conclusions

This study investigates two aminocoumarin derivatives (102 and 153) with distinctive ICT characteristics to elucidate the mechanisms governing their stimulated emission and nonlinear absorption properties. FMO and NTO analyses visualize electronic transitions, identifying dominant local excitation and enhanced ICT features. Transient dynamics analysis reveals that enhanced ICT induces a red shift in the stimulated emission band. At 515 nm, femtosecond Z-scan measurements demonstrate RSA-to-SA switching behavior. This work demonstrates the ICT-mediated control of stimulated emission and nonlinear absorption in coumarin systems as a fundamental structure–property relationship. Our findings position these coumarin derivatives as enabling materials for reconfigurable photonic devices, as their nonlinear response can be switched between RSA and SA simply by modifying the electron-withdrawing group. This tunability enables novel applications such as smart optical limiters with dual-threshold operation—where SA allows for a high transmission at low fluence for clear imaging, while RSA provides protection under high-intensity pulses—and self-activating Q-switches that offer built-in protection against stray light without additional components. Such molecular-level control permits functionality to be designed directly into photonic device layers during fabrication, facilitating advanced integrated systems with tailored optical behavior.

## Figures and Tables

**Table 1:** Calculated dipole moments of ground state S_0_ (μ_00_) and excited state S_1_ (μ_11_), transition dipole moment (μ_01_) and static polarizability (α(0)), and second-order static hyperpolarizability (γ(0,0,0)) for coumarin 102 and 153.

Sample	μ_00_ (a.u.)			μ_01_ (a.u.)				μ_11_ (a.u.)			α(0) a.u.	γ(0,0,0) ×10^5^ a.u.
	X	Y	Z	X	Y	Z	*f* *	X	Y	Z		
102	3.908	2.160	0.161	−2.480	−0.198	−0.031	0.493	8.629	1.440	0.180	77.214	2.527
153	−4.317	−1.976	0.103	2.355	0.942	−0.050	0.460	−9.527	−3.043	0.170	83.973	4.204

* Oscillator strengths of transition dipole moment μ_01_.

**Table 2:** Z-scan fitting parameters for coumarins 102 and 153.

Sample	*α*_0_ (m^−1^)	*β* (m/W)	*I_c_* (GW/cm^2^)
Coumarin 102	19.46	1.00 × 10^−13^	-
Coumarin 153	91.00	1.34 × 10^−13^	9.8

## Data Availability

Dataset available on request from the authors.

## Supplementary Materials

The following supporting information can be downloaded at: https://www.mdpi.com/article/10.3390/molecules30183799/s1, Figure S1: Fluorescence decay kinetics recorded at 380 and 430 nm for coumarin 102 (a) and 153 (b). Figure S2: Open-aperture Z-scan experiments of coumarin 153 at 515 nm with different input light intensity. Synthetic protocols for coumarin 102 and 153.

## Abbreviations

The following abbreviations are used in this manuscript:

NLO	Nonlinear optical
ICT	Intramolecular charge transfer
RSA	Reverse saturable absorption
SA	Saturable absorption
NLA	Nonlinear optical absorption
SAs	Saturable absorbers
OLs	Optical limiters
DMSO	Dimethyl sulfoxide
FMO	Frontier molecular orbital
NTO	Natural transition orbital
TAS	Transient absorption spectroscopy
DFT	Density functional theory
TD-DFT	Time-dependent density functional theory
OPA	Optical parametric amplifier
