# Supplementary material for: Controlling Stimulated Emission via Intramolecular Charge Transfer in Amino-Coumarin Dyes: Switching from Reverse Saturable to Saturable Absorption
Source: Molecules. 2025 Sep 18;30(18):3799. doi: 10.3390/molecules30183799 (PMC12472746; doi:10.3390/molecules30183799)
Supplement: Supplementary file 1 [file molecules-30-03799-s001.zip › molecules-3804534-supplementary.pdf]

Supporting Information

1. Time-Resolved Fluorescence Decay

To investigate the effect of intramolecular charge transfer (ICT) on the fluorescence emission of amino-coumarins 102 and 153, time-resolved fluorescence decay measurements were conducted. We observed that the fluorescence decay kinetics of coumarin 102 differ significantly from those of coumarin 153. The decay curve of coumarin 102 exhibits a monoexponential profile with a lifetime τ of 0.424 ns. In contrast, the decay of coumarin 153 follows a biexponential behavior, with lifetimes τ_1 = 1.248 ns and τ_2 = 4.280 ns. This marked difference in photophysical behavior can be attributed to the enhanced ICT character in coumarin 153.

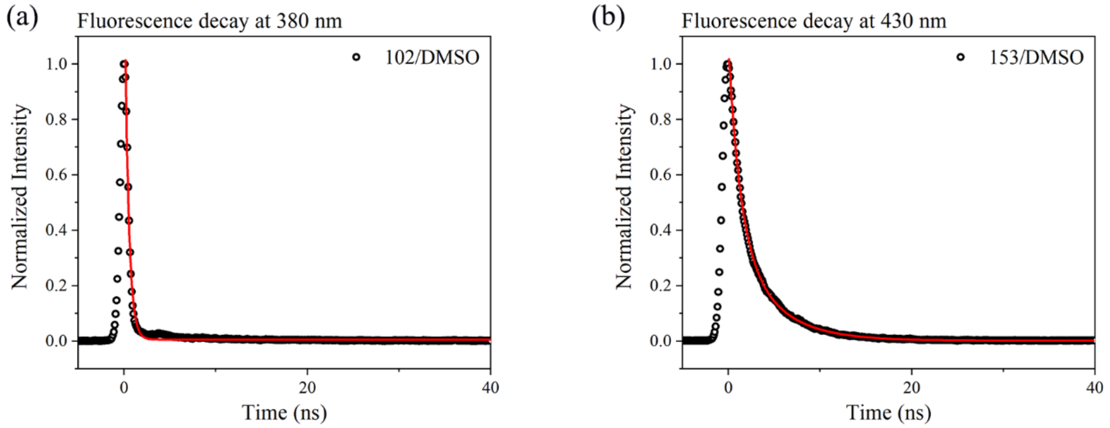

Figure S1. Fluorescence decay kinetics recorded at 380 and 430 nm for coumarin 102 (a) and 153 (b).

2. Fluorescence Lifetime

We performed the open-aperture Z-scan experiments of coumarin 153 at 515 nm with different excitation light intensities, as shown in **Figure S2**. At an incident intensity of 84.5 GW/cm^2, SA stemming from stimulated emission is the dominant effect. However,

the intensity threshold at which RSA from excited-state absorption becomes dominant could not be determined, as it exceeds the maximum available intensity of 93.7 GW/cm² from our light source.

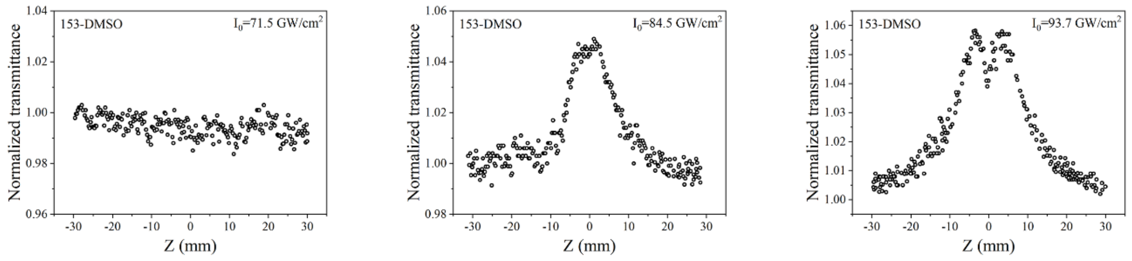

Figure S2. Open-aperture Z-scan experiments of coumarin 153 at 515 nm with different input light intensity.

3. Synthesis of coumarin 102 and 153

Synthesis of Coumarin 102:

A mixture of chlorot(triphenylphosphine)gold(I) ($Au(PPh_3)Cl$, 10 mg, 0.02 mmol) and silver hexafluoroantimonate ($AgSbF_6$, 7 mg, 0.02 mmol) was dissolved in a 1:1 mixture of dichloroethane (DCE) and 1,4-dioxane (5 mL) in an 8 mL vial. To this solution was added a solution of 2,3,6,7-tetrahydro-1H,5H-benzo[ij]quinolizin-8-yl 2-butynoate (137 mg, 0.4 mmol) in the same solvent mixture (3 mL). The vial was sealed under air and stirred at room temperature, with the reaction progress monitored by TLC. Upon completion, the solvent was removed under reduced pressure, and the crude residue was purified by column chromatography on silica gel, eluting with a mixture of dichloromethane and ethyl acetate (80:20), to afford the title compound as a pure product in 91% yield.

Synthesis of Coumarin 153:

A mixture of 8-hydroxyjulolidine (1.0 g, 5.28 mmol), ethyl 4,4,4-trifluoroacetoacetate (1.17 g, 6.34 mmol), and anhydrous zinc chloride (0.5 g) as a catalyst in anhydrous ethanol (20 mL) was heated at 60 °C for 6 hours. After the reaction was complete (monitored by TLC), the mixture was cooled to room temperature. The solvent was then removed under reduced pressure, and the crude residue was purified by column chromatography on silica gel to afford the title compound as a pure product in 72% yield.
